# In-Situ Synthesis of Hydrophobic Polyurethane Ternary Composite Induced by Hydroxyethyl Cellulose through a Green Method for Efficient Oil Removal

**DOI:** 10.3390/polym12030509

**Published:** 2020-02-26

**Authors:** Junyong Chen, Xian Yue, Zhou Xiao, Huaxin Li, Xianbo Yu, Junhui Xiang

**Affiliations:** Center of Materials Science and Optoelectronic Engineering, College of Materials Science and Optoelectronic Technology, University of the Chinese Academy of Sciences, Beijing 100049, China; chenjunyong17@mails.ucas.ac.cn (J.C.); lexian16@mails.ucas.ac.cn (X.Y.); xiaozhou19@mails.ucas.ac.cn (Z.X.); lihuaxin17@mails.ucas.ac.cn (H.L.); yuxianbo18@mails.ucas.ac.cn (X.Y.)

**Keywords:** aqueous dispersion polymerization, induced self-assembly, hydrophobicity, oil-water separation

## Abstract

Hydroxyethyl cellulose (HEC) was introduced to activate the surface of polyurethane (PU) sponge to successfully prepare a hydrophobic ternary composite PU/HEC/SiO_2_. The hydrophobic layer of the composite was realized by in-situ polymerization of methyltriethoxysilane (MTES) onto the surface of PU sponge. The formation of a stable hydrophobic SiO_2_ layer solved successfully the problem of ease of SiO_2_ particles shedding from the composite. Moreover, the amphiphilic molecules produced by the hydrolysis of MTES monomers facilitated the preparation of hydrophobic materials by aqueous dispersion polymerization. Aqueous synthesis made the reaction process environmentally-friendly and pollution-free. The as-prepared composite PU/HEC/SiO_2_ not only retains high porosity and low density of the PU sponge, but also considerably reduced the surface free energy and increased the surface roughness of the PU sponge. Therefore, outstanding hydrophobicity and high porosity endow the composite with excellent oil removal capability as a high-efficiency absorbent. Moreover, the hydrophobic composite that had absorbed oil could be regenerated easily by squeezing and recycling.

## 1. Introduction

With the development of offshore oil exploitation and ocean transportation, marine pollution accidents involving petroleum occur frequently, seriously destroying the environment and posing a threat to human health [1]. Therefore, controlling oil pollution is an urgent and challenging task. Traditional oil-water separation strategies, such as biodegradation, precipitation, and combustion, have been used in oil pollution control [2]. However, these traditional technologies are usually inefficient and can easily cause secondary pollution to the environment [3]. Absorption technology is considered to be one of the most effective pollution control methods because it is relatively efficient and easy to operate [4]. 

To date, commercial resins [5,6,7], fibers [8,9,10], mineral products [11,12], and other absorption materials [13,14] are deemed cheap and easy to obtain, and have been developed and widely used in oil-water separation applications. However, these traditional absorbents have some shortcomings, such as poor selectivity, low absorptivity, difficulty in large-scale manufacturing, and complex subsequent processing procedures. Therefore, researchers are trying to explore better new oil-water separation materials. 

Recently, some new and efficient absorbent materials such as photoresponsive materials [15], carbon nanotubes (CNTs) [16], graphene-based materials [17,18,19], nanocellulose materials [20,21,22,23,24], and PU-based absorbents have attracted significant attention in oil-water separation applications [25,26]. PU sponge is considered to be one of the best absorbent methods because of its low density, high porosity, and high elasticity [27,28,29]. However, polyurethane (PU) is an amphiphilic material and does not possess the ability to separate an oil-water mixture. Therefore, hydrophobic modification of PU sponge is needed for selective separation.

According to the Cassie–Baxter model [30,31,32], the wettability of materials depends on the surface roughness and surface free energy. Therefore, the main approach to surface hydrophobic modification are to reduce surface energy and structure micro-roughness. There are several common methods for surface modification of PU. Chemical deposition [33], wet chemical reaction [34,35], polymerization [36,37], electroless deposition [38], and carbonization [39] are some of the methods. Among these, wet chemical reactions have the advantages of mild conditions and simple operation. Vintu and Unnikrishnan obtained a type of indolocarbazole-based polymer-coated super absorbent PU sponges by the Sonogashira coupling reaction [40]. However, in the synthesis of the complex material, large amounts of organic solvents (e.g., trichloromethane and cyclohexane) had been used, which caused complex post-processing safety and pollution problems. Yuan et al. reported an organic-inorganic composite—hierarchical hollow SiO_2_@MnO_2_ cube-reinforced elastic PU foam for oil-water saperation [41]. However, these nanoparticles containing heavy metals (Mn^4+^) are inclined to move away from the surface of the complex during long-term utilization process, causing secondary pollution.

Herein, we designed a novel PU/HEC/SiO_2_ composite by a two-step impregnation strategy, using two environment-friendly materials hydroxyethyl cellulose (HEC) and methyltriethoxysilane (MTES) as precursors. In the ternary PU/HEC/SiO_2_ composite, the introduction of HEC can induce MTES to grow on the surface of PU and form a stable hydrophobic SiO_2_ layer, thus solving the problem of ease of SiO_2_ nanoparticles shedding from the composite by forming a silicon-oxygen bond. Remarkably, the hydrophobic composite was synthesized in aqueous phase rather than organic solvents. The as-prepared composite exhibited excellent hydrophobicity and oleophilicity. Furthermore, The PU/HEC/SiO_2_ composite could not only remove oil from water rapidly and efficiently, but could also be reused. In summary, we provide a facile and green strategy to prepare the ternary PU/HEC/SiO_2_ composite, which has potential applications in the field of oil-water separation. 

## 2. Experimental Section

### 2.1. Materials

The reagents, including methyltriethoxysilane (MTES, ≥98%), hydrochloric acid (HCl, AR) and sodium hydroxide (AR) were bought from Sinopharm Chemical Regent Co., Ltd (Shanghai, China). Sudan III and Methyl blue were purchased from Sigma Chemistry Co. Ltd (Shanghai, China). Organic solvents, including dichloromethane (AR), chloroform (AR), n-hexane (AR), toluene (AR), and chlorobenzene (AR) were purchased from Beijing Chemical Reagents Co. (Beijing, China). Gasoline was obtained from Sinopec Corp. Hydroxyethyl Cellulose was purchased from Aladdin–Holdings Group (Shanghai, China). Commercial PU sponge was supplied by Alibaba Enterprise (Hangzhou, China). 

### 2.2. Preparation of Binary PU/HEC Composite

The PU/HEC composite was prepared by impregnation method. 0.3 g hydroxyethyl cellulose was dissolved in 50 mL deionized water. Magnetic stirring was performed at room temperature for 1 h to form a uniform water solution. Then PU (with a size of 2 × 2 × 2 cm^3^) sponge was immersed in the above water solution, stirred for 5 min, and dried at 50 °C to obtain the PU/HEC composite sample.

### 2.3. Preparation of Ternary PU/HEC/SiO_2_ Composite

**Figure 1:**
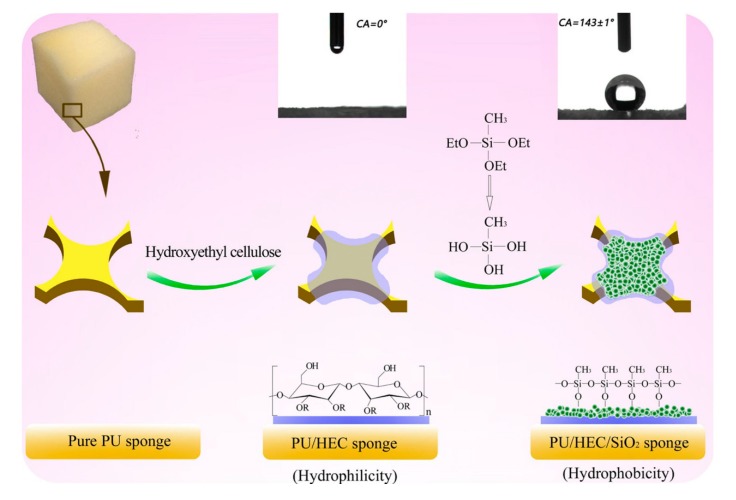
Schematic illustration of HEC-induced self-assembly synthesis of the composite PU/HEC/SiO_2_. Abbreviations: HEC, hydroxyethyl cellulose; PU, polyurethane.

Firstly, 2 mL MTES, with 22.4 mL deionized water were added into a beaker successively, and 0.2 mL hydrochloric acid catalyst (*w*/*w* 1% in H_2_O) was added into the flask within a minute, then stirred for 0.5 h. Sodium hydroxide (1 mol/L in H_2_O) was added rapidly to adjust the solution pH value to 10, and then kept stirring for a minute. Secondly, we let the re-prepared dried PU/HEC composite soak for a minute in the solution mentioned above. Then, wet sample was pulled out of the liquid phase, and then aged at 50 °C for 3 h. Subsequently, the sample was washed with de-ionized water for several times. Finally, the sample was freeze-dried to get ternary PU/HEC/SiO_2_ composite. The preparation process and mechanism of the ternary composite was shown schematically in Figure 1. Firstly, HEC, rich in a large number of hydroxyl groups, was introduced to the material PU surface. Hydroxyl groups of the surface provided a chemical basis for coating of MTES on the surface. MTES could be hydrolyzed to silanol molecules with the existence of acid catalyst. Furthermore, the forming silanol molecules could react with hydroxyl groups of the surface of the composite PU/HEC, and more silanol molecules induced to in-situ polymerize to the surface of the composite to form SiO_2_ layer [42].

### 2.4. Characterization

The morphologies of different samples were investigated by a field-emission scanning electron microscope (SEM, S-4800 Hitachi, Tokyo, Japan). The Fourier transform infrared (FTIR) spectra of the samples were obtained by a VERTEX 70 FTIR spectrometer (Bruker, Ettlingen, Germany). Static contact angles of different samples were performed through a contact angle meter (JC2000C1, Powereach, Shanghai, China). In the measurements of static contact angle, three parallel samples were conducted in each case and five measurements were taken for each sample.

### 2.5. Oil-Water Separation Experiments

The prepared PU/HEC/SiO_2_ sample was cut into sample with a size of 2 × 2 × 2 cm^3^. The separation experiments for the oil-water mixture were then carried out. Samples were placed in oil-water mixture for one minute to absorb the oil until they were completely saturated and then removed for weighing. The oil absorption properties of the composite were tested by comparing the mass changes of the composite before and after oil absorption. A composite sample with a mass of *M*_0_ was placed in the container containing oil. After absorbing the oil to saturation, the sample was taken out and the weight recorded as *M_t_*. The oil absorption rate is calculated according to Equation (1):

*C*_0_ (g/g) = (*M_t_* − *M*_0_)/*M*_0_ (1)

where *M*_0_ and *M_t_* are the weights of the PU/HEC/SiO_2_ before and after oil uptake, respectively. 

According to the references [25,28], the amount of residula oil can be calculated through simple gravimetric methods. Five grams of given oil was added into 20 mL water to form the oil-water mixture (*M_mix,_*_0_). Then the composite sample was placed at the oil-water interface for one minute. After the composite was taken out, the oil-water mixture was weighed again (*M_mix,t_*). The separation efficiency (*f*%) was calculated according to Equation (2):

*f* (%) = (*M_mix,_*_0_ − *M_mix,t_*)/*M_oil_* × 100% (2)

where *M_oil_* represents the weight of the given oil (5 g).

Then, the amount of residual oil in the water phase (*R*%) was calculated using Equation (3).

*R* (%) = (100 − *f)* × 100% (3)

## 3. Results and Discussion

### 3.1. Morphological Characterization

**Figure 2:**
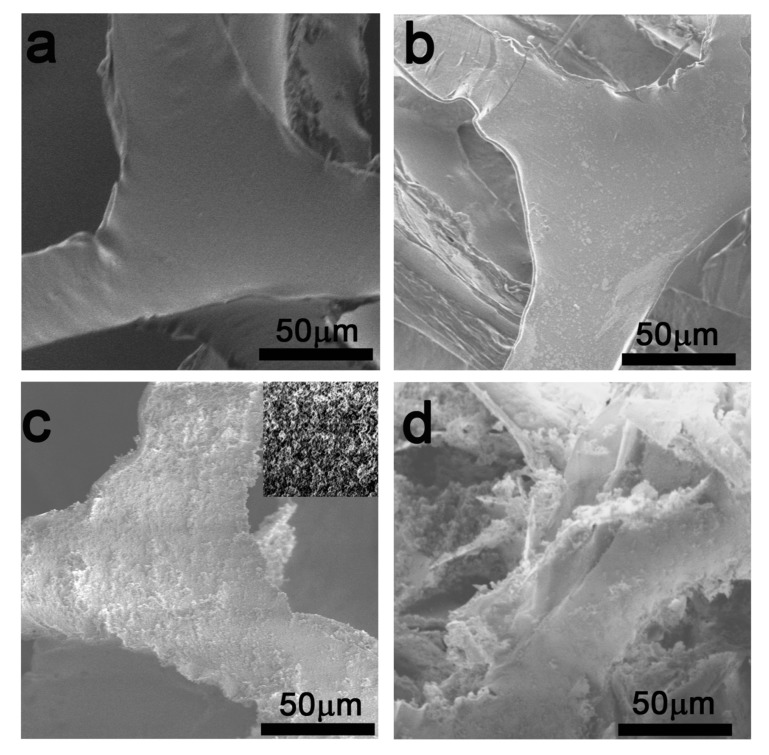
SEM images of unmodified PU sponge (**a**), HEC-modified PU composite PU/HEC (**b**), HEC- and MTES-modified PU composite PU/HEC/SiO_2_ (the inset is its local enlarged image) (**c**), MTES-modified PU composite PU/SiO_2_ (**d**). Abbreviations: MTES, methyltriethoxysilane.

**Figure 3:**
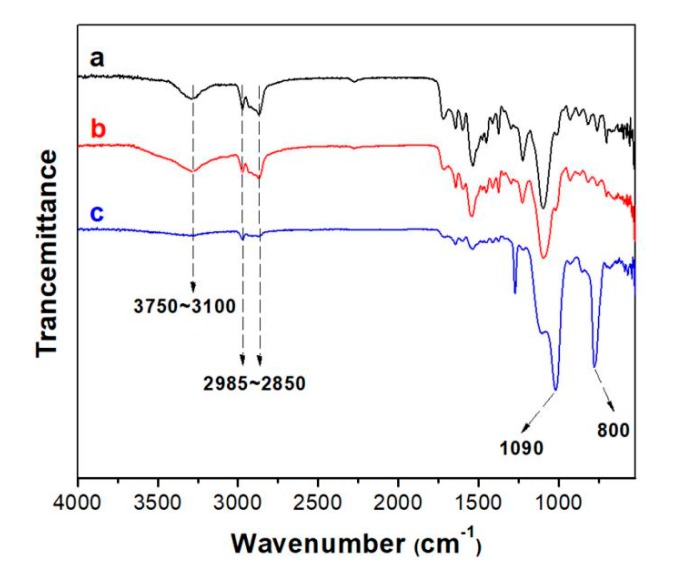
Fourier transform infrared (FTIR) spectra of PU (**a**), PU/HEC (**b**), and PU/HEC/SiO_2_ (**c**).

The microtopography images of the pure PU and PU/HEC samples were shown in Figure 2a,b, respectively. It was clear that the introduction of HEC slightly changes the surface roughness of the PU surface. Meanwhile, the cracks during the making of SEM samples (in the upper left corner of Figure 2b) and the FTIR spectra of PU/HEC sample also proved that HEC was successfully modified in-situ on the surface of PU (Figure 3b). In addition, we could regulate the quantities of HEC to control the coverage of HEC on the PU surface. Hence, an experiment was carried out with different dosages of HEC, namely, 0.1, 0.3, 0.5, and 0.7 g. It was found that the ratio of HEC coating onto PU improved gradually with increasing dosage of HEC, as shown in Figure S1a–c. Even when the dosage was 0.3 g, some pores of PU were significantly blocked by the HEC layer (Figure S2d). The appropriate dosage of HEC was 0.3 g according to the experimental results. It can be seen from the SEM images that the PU/HEC (Figure 2b) and PU/HEC/SiO_2_ (Figure 2c) samples retained the three-dimensional skeleton structure of the PU sponge (Figure 2a). SiO_2_ nanoparticles were successfully modified on the surface of PU, and the particle sizes were less than 100 nm (Figure S3). This indicates that a rough composite network structure was formed on the surface of the PU sponge after a two-step impregnation. In addition, the influence of the quantities of MTES on the surface coverage of the composite was investigated within a certain range of dosages (i.e., runs of 1, 2, 3 and 4 mL). When the amount of MTES was insufficient, in-situ polymerization of MTES on the composite surface occurred preferentially because of the inductive effect of HEC, as shown in Figure S2a,b. When the dosage of MTES is too high, the nucleation, growth, and aggregation of excessive silanol molecules (MTES hydrolysate) also began in the composite pores, leading to the blockage of the pores, as shown in Figure S2c,d. After optimization, we finally chose an MTES dosage of 2 mL. In contrast, without HEC, the PU surface could not provide abundant reactive sites; most of the SiO_2_ nanoparticles were only filled in the structural cavities, rather than covering the surface of the PU three-dimensional skeleton. Therefore, the pores structure of the sponge was seriously destroyed, as shown in Figure 2d. During the use of the PU/SiO_2_, SiO_2_ composite, SiO_2_ particles could easily fall off from PU, which weakened the repeated use stability of the composite. Through comparing Figure 2c,d, the importance and validity of HEC has been firmly established. As the surface activating material, HEC can induce self-assembly of silanol molecules to the surface of the composite to form SiO_2_ layer. 

### 3.2. FT-IR Spectra

The surface functional groups of PU/HEC and PU/HEC/SiO_2_ were analyzed by FTIR, as shown in Figure 3. The wide absorption band in Figure 3a at 3200 cm^−1^ corresponds to the N–H of PU sponge. In comparison, the characteristic peak of PU/HEC at 3750~3100 cm^−^^1^ (Figure 3b) increased significantly, because O–H stretching vibration of HEC significantly enhanced the signal strength [43]. Moreover, the wave number between 2985~2850 cm^−1^ corresponded to C–H stretching vibration in all samples, which also proved the existence of the methyl group of PU/HEC/SiO_2_ [12]. PU/HEC/SiO_2_ exhibited strong infrared absorption peaks at 800 and 1090 cm^−1^, respectively, which corresponded to the stretching vibration characteristic peaks of Si–O (Figure 3c) [44]. In Figure 3c, the disappearance of O–H and appearing of Si–O demonstrated that MTES and PU/HEC formed a stable structure through oxygen and silicon bonds, instead of van der Waals forces. The analysis of FTIR spectra proved that MTES was successfully modified on the surface of PU/HEC to form a hydrophilic ternary composite PU/HEC/SiO_2_.

### 3.3. Wettability

**Figure 4:**
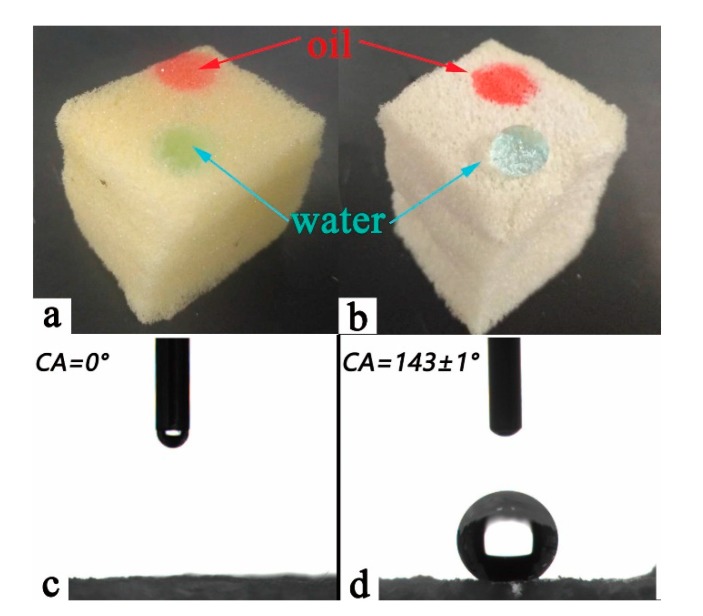
Water (colored blue) and oil (colored red) dropped onto the surface of PU/HEC (**a**) and the PU/HEC/SiO_2_ (**b**). WCAs of pure PU/HEC (**c**) and the PU/HEC/SiO_2_ (**d**). The plant oil was colored red with Sudan Ⅲ while water was colored blue with Methyl blue prior to the experiment.

As shown in Figure 4, the water contact angle of PU/HEC material was 0° (Figure 4c), while the water contact angle of the composite PU/HEC/SiO_2_ reached 143 ± 1° (Figure 4d). When the surface of PU was covered with HEC, a large amount of hydroxy was exposed on the surface of PU. Owing to hydroxy is a hydrophilic group, the binary composite PU/HEC showed hydrophilic and lipophilic properties (Figure 4a). However, MTES-modified PU/HEC/SiO_2_ composite possessed a low surface free energy and suitable surface roughness, which were two key factors for materials with special wettability according to the Cassie–Baxter model [30,31,32]. Hence the composite exhibited excellent hydrophobic property (Figure 4b).

### 3.4. Oil-Water Separation Process

**Figure 5:**
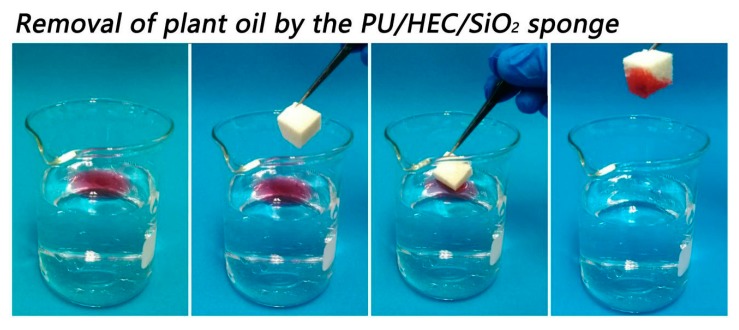
Selected removal of plant oil from water with the PU/HEC/SiO_2_ composite. Plant oil was colored red with Sudan Ⅲ prior to the experiment.

In order to verify the selective absorption ability, the composite was applied to the separation of oil-water mixture. The oil-water separation process of the composite was shown in Figure 5. Plant oil (dyed with Sudan Ⅲ) was spread over the water surface to form oil- water mixture. The PU/HEC/SiO_2_ composite was placed in the oil/water mixture. The sample quickly and selectively removed the plant oil, leaving only clean water. The results demonstrated that the composite was an efficient absorbent for oil-water separation.

### 3.5. Recyclability of the Ternary PU/HEC/SiO_2_ Composite

**Figure 6:**
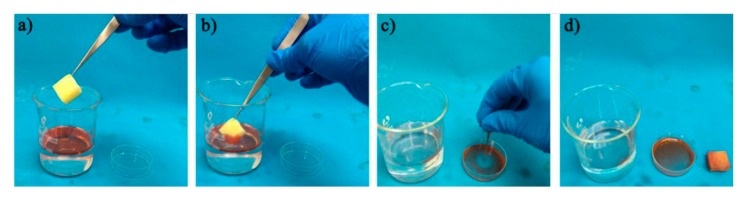
Oil absorption-desorption process of the PU/HEC/SiO_2_ composite. (**a**) Before absorption. (**b**) Absorption in progress. (**c**) Desorption progress by squeezing. (**d**) After desorption. The plant oil was colored red with Sudan Ⅲ prior to the experiment.

**Figure 7:**
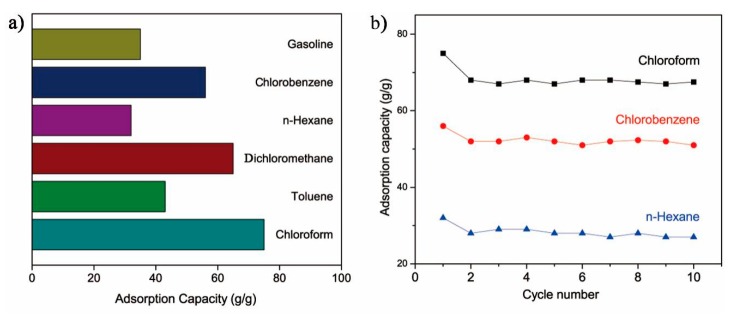
Oil removal tests of the PU/HEC/SiO_2_. (**a**) Maximum absorption capacities towards six different types of organic solvents. (**b**) Investigation of the reusability of the composite towards several organic solvents for 10 cycles.

In order to verify the recyclability of the composite, we examined the processes of absorption, desorption, and regeneration of the composite, as shown in Figure 6. Firstly, the composite was placed on the surface of the oil-water mixture. The composite immediately absorbed the plant oil (colored red with Sudan III), leaving the water (Figure 6b). The hydrophobic composite that had absorbed oil could be regenerated easily by squeezing out the oil and recycling the composite, a simple process to operate; the process is high-performing and cost-effective (Figure 6c). After the first regeneration, some residual oil remained in the composite (Figure 6d), but it had negligible effect on the recycling performance, as shown in Figure 7b [43].

### 3.6. Absorption Amount

In order to measure the oil absorption capacity of the PU/HEC/SiO_2_ composite, a variety of oils and organic solvents were selected to test the oil absorption capacity. All the results are shown in Figure 7. The results showed that the absorption capacity of the composite for various oils could reach 36–75 times the sample weight. The difference in oil absorption capacity of different oil products was mainly caused by the different densities of the oil products. In addition, the composite possessed a comparatively high absorption capacity for organic solvents of high density. After 10 recycling cycles, the oil-removal capacity of the composite remained basically unchanged with a modest decrease. We concluded that there are two main reasons for the decrease in the water contact angle: (1) it is probably caused by residual oils in the pores of the composite after the first regeneration, which cannot be removed by manual squeezing [45]; (2) during the process of oil desorption, the SiO_2_ layer of the composite may decrease slightly and affect the hydrophobic performance of the composite. However, it is difficult to find obvious changes in the surface morphology after repeated squeezing (Figure S4). 

Gasoline is one of the most common fuels in human production and life. Using gasoline as the representative, we investigated the oil-water separation efficiency of the PU/HEC/SiO_2_ composite. The result proved that the composite possessed excellent separation efficiency and the amount of residual oil in the water phase could be controlled under 2%. Results showed that the as-prepared composite could be used as an efficient absorbent for the oil-water separation.

### 3.7. Acid and Alkali Resistance

**Figure 8:**
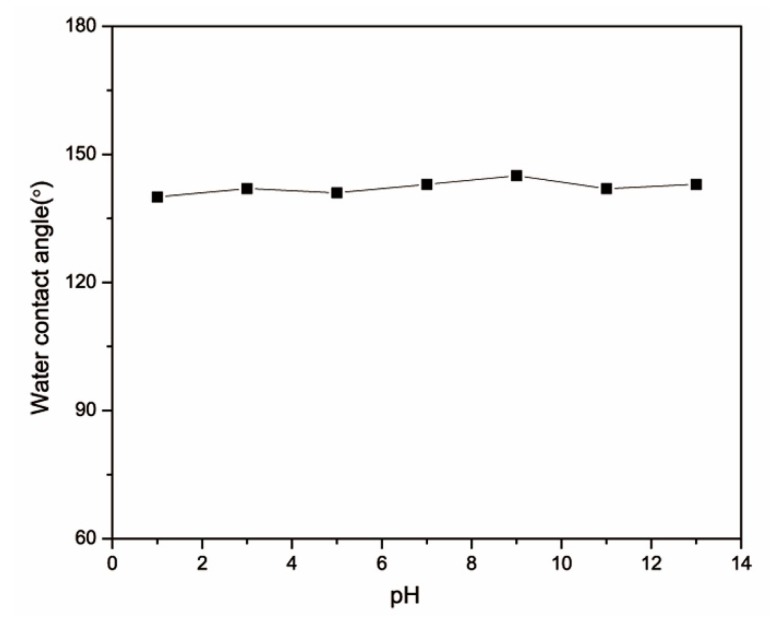
Water contact angles of the PU/HEC/SiO_2_ composite with different pH values.

In the practical application of oil-water separation, the acid and alkali resistance of materials is also a very important technical index to measure the performance of the absorbent. Herein, the effect of different pH values on the hydrophobic properties of the composite was investigated. Different pH values (pH = 1–13) of water solution were added to the composite surface, and the contact angle was measured after a 1 h interval. All the experimental results were shown in Figure 8. The experimental results showed that the composite also possessed excellent hydrophobic properties at different pH values, demonstrating its excellent acid and alkali resistance.

## 4. Conclusions

In summary, two environment-friendly materials, HEC and MTES, were used as hydrophobic surface treatment for the PU sponge to successfully prepare a flexible and hydrophobic ternary PU/HEC/SiO_2_ composite. HEC induced in-situ polymerization of MTES on the surface of the composite, resulting in chemical interaction and the formation of a stable structure through the oxygen and silicon bonds. Moreover, instead of various organic solvents, water was used as the sole solvent in the two-step synthesis process, which was green and environmentally-friendly. The as-prepared PU/HEC/SiO_2_ composite not only retained the high porosity and low density of the PU sponge, but also reduced the surface free energy and increased the surface roughness of PU. Reducing the surface free energy and increasing the roughness can considerably improve the material hydrophobicity according to the Cassie–Baxter theory. The water contact angle of the composites reached 143 ± 1° by measurement. Oil absorption experiments showed that the composite had a high oil absorption capacity and could absorb up to 36–75 times its own weight. The hydrophobic composite that has absorbed oil could be regenerated easily by squeezing and recycling. Moreover, after repeated 10 oil-water separation cycles, the composite still maintained excellent hydrophobicity and oil removal ability, which fully demonstrate the excellent durability of the composite. We concluded that SiO_2_ and PU could form a stable ternary composite system under the induction of HEC, which effectively prevented SiO_2_ nanoparticles from falling off the PU surface. These results fully prove that the ternary PU/HEC/SiO_2_ composite has significant potential for application in the field of oil-water separation.

## Supplementary Materials

The following are available online at https://www.mdpi.com/2073-4360/12/3/509/s1, Figure S1: SEM images of the composite PU/HEC with different dosages of HEC. 0.1 g (a); 0.3 g (b); 0.5 g (c); 0.7 g (d), Figure S2: SEM images of the composite PU/HEC/SiO_2_ with different dosages of MTES. 1 mL (a); 2 mL (b); 3 mL (c); 4 mL (d), Figure S3: SEM image of the composite PU/HEC/SiO_2_, Figure S4: SEM image of the composite PU/HEC/SiO_2_ after repeated squeezing (runs 10 times).
