# Supplementary material for: In-Situ Synthesis of Hydrophobic Polyurethane Ternary Composite Induced by Hydroxyethyl Cellulose through a Green Method for Efficient Oil Removal
Source: Polymers (Basel). 2020 Feb 26;12(3):509. doi: 10.3390/polym12030509 (PMC7182898; doi:10.3390/polym12030509)
Supplement: Supplementary file 1 [file polymers-12-00509-s001.pdf]

Supporting information

In-situ synthesis of hydrophobic polyurethane ternary composite induced by hydroxyethyl cellulose through a green method for efficient oil removal

Junyong Chen, Xian Yue, Zhou Xiao, Huaxin Li, Xianbo Yu and Junhui Xiang *

Center of Materials Science and Optoelectronic Engineering, College of Materials Science and Optoelectronic Technology, University of the Chinese Academy of Sciences, Beijing 100048, China; chenjunyong17@mails.ucas.ac.cn (J.C.); lexian16@mails.ucas.ac.cn (X.L.); xiaozhou19@mails.ucas.ac.cn (Z.X.); lihuaxin17@mails.ucas.ac.cn (H.L.); yuxianbo18@mails.ucas.ac.cn (X.Y.)

* Correspondence: xiangjh@ucas.ac.cn

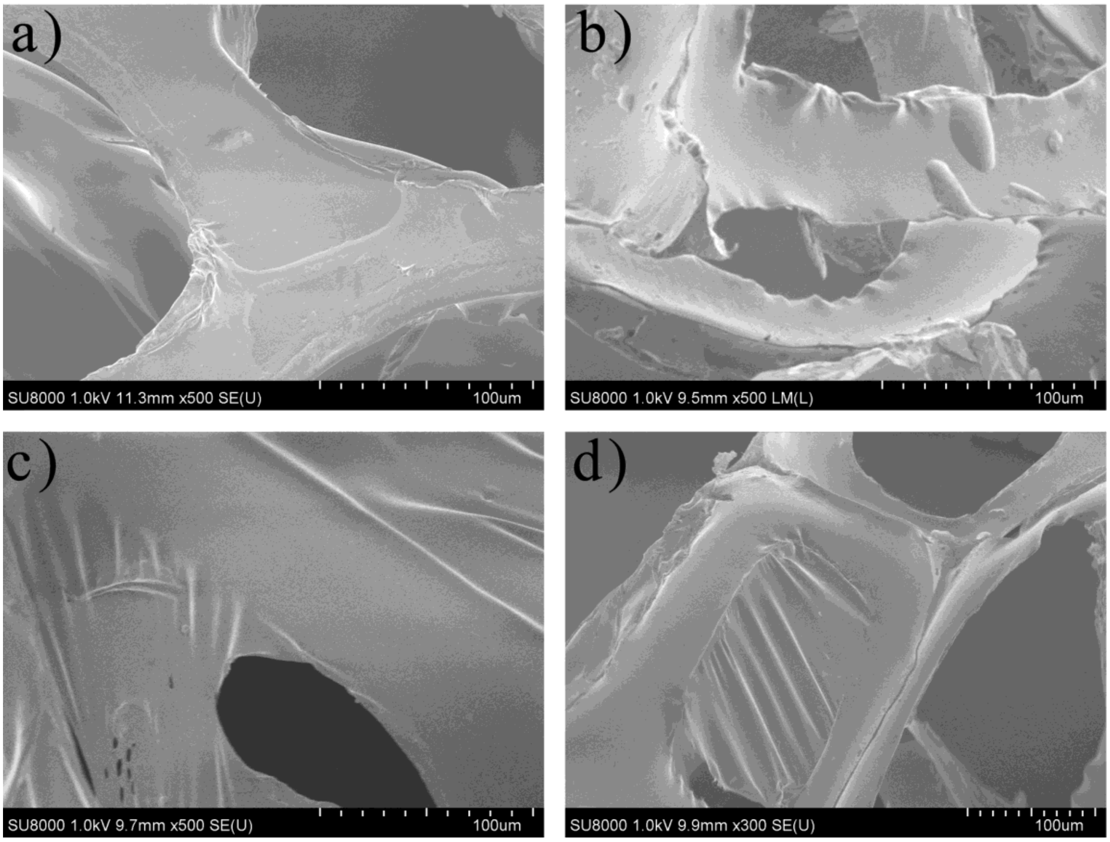

Figure S1. SEM images of the composite PU/HEC with different dosages of HEC. 0.1g (a); 0.3g (b); 0.5g (c); 0.7g (d).

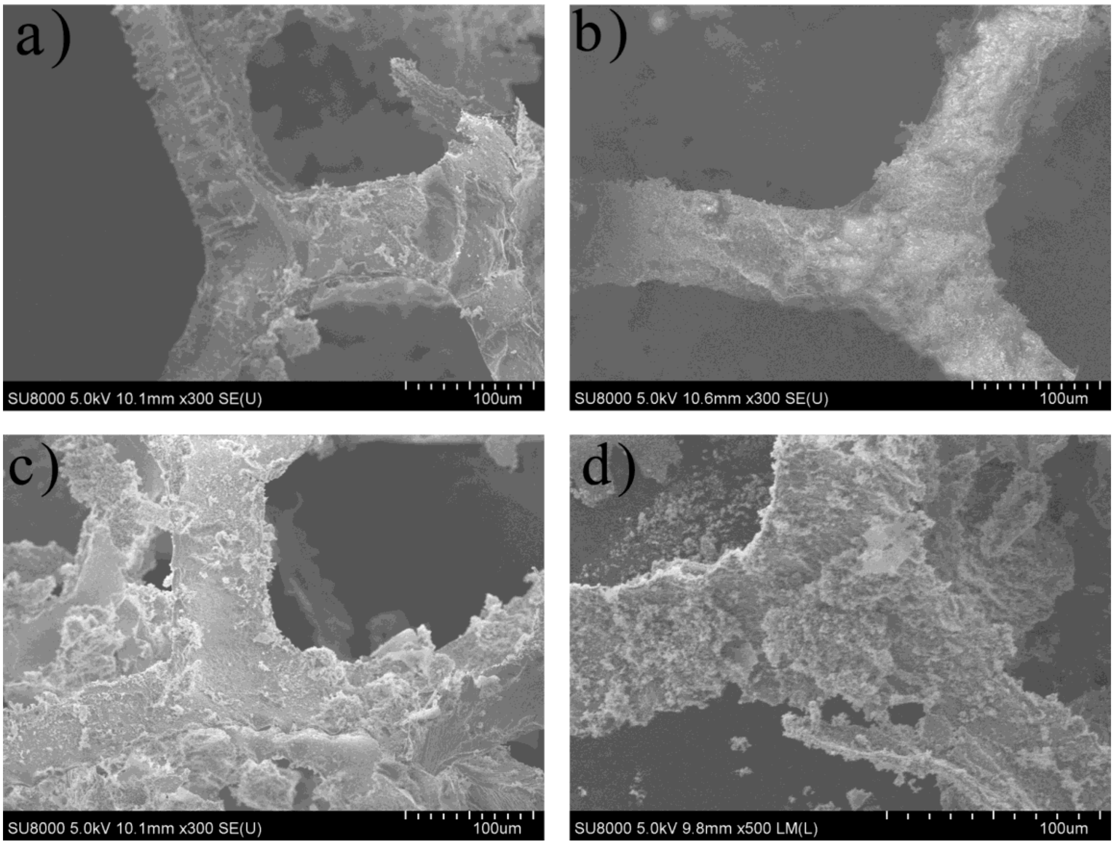

Figure S2. SEM images of the composite PU/HEC/SiO_2 with different dosages of MTES. 1mL (a); 2mL (b);3mL (c);4mL (d).

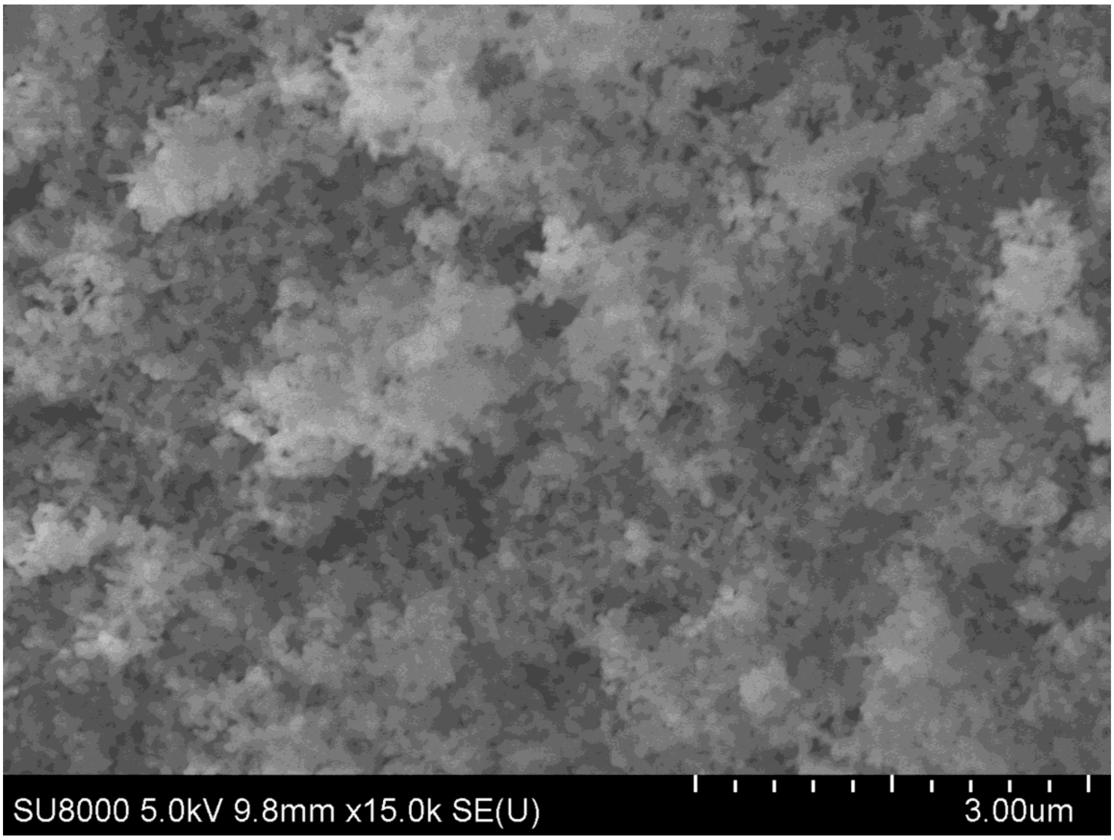

Figure S3. SEM image of the composite PU/HEC/SiO_2.

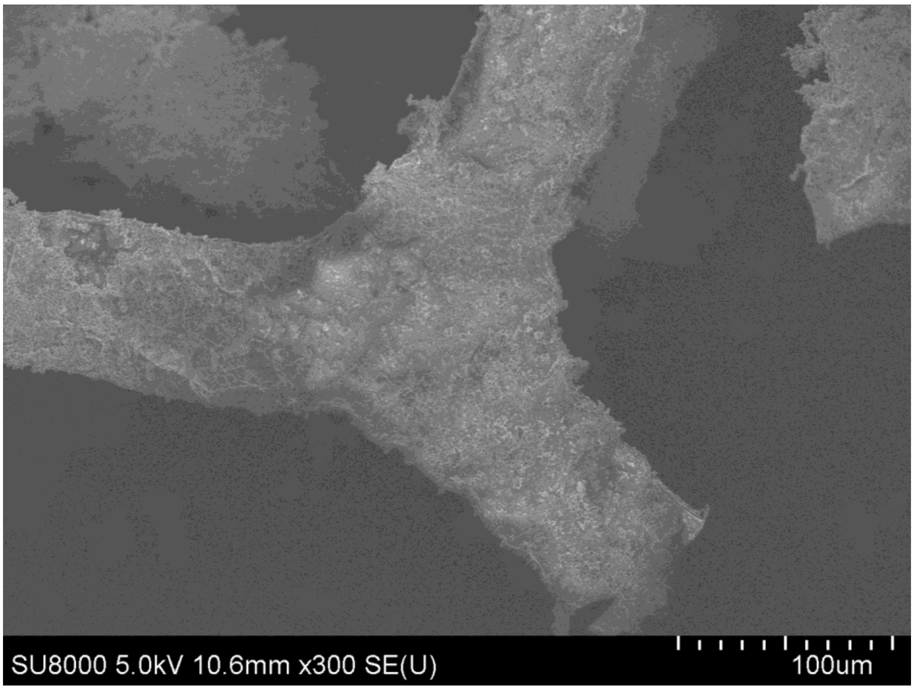

Figure S4. SEM image of the composite PU/HEC/SiO_2 after repeated squeezing (runs 10 times).
